# Head and Neck Radiotherapy Short-Term Oral Complications: Effect on Oral Health-Related Quality of Life at Dharmais Cancer Hospital

**DOI:** 10.1055/s-0046-1816079

**Published:** 2026-02-12

**Authors:** Mohamad Zulfikar Idris, Yuniardini Septorini Wimardhani, Widya Apsari, Masita Mandasari

**Affiliations:** 1 Oral Medicine Residency Program, Faculty of Dentistry, Universitas Indonesia, Jakarta, Indonesia; 2 Oral Medicine Department, Faculty of Dentistry, Universitas Indonesia, Jakarta, Indonesia; 3 Oral Medicine Division, Department of Dentistry, Dharmais Cancer Hospital, Jakarta, Indonesia

**Keywords:** head and neck cancer, radiotherapy, oral complications, quality of life, OHIP-14

## Abstract

**Objective:**

To describe the effect of short-term oral complications of head and neck cancer (HNC) radiotherapy on patients' quality of life (QoL). Radiotherapy is one of the most common HNC therapy modality, either as the primary therapy or in combination with others. However, high-dose radiotherapy can cause both short- and long-term oral complications. Short-term oral complications of radiotherapy include oral mucositis, hyposalivation, dysphagia, and sensory disturbances which can affect the patients' ability to speak, chewing, and swallowing, leading to weight loss and a decline in QoL, thus delaying the therapy process.

**Materials and Methods:**

This is a cross-sectional study at the Dharmais Cancer Hospital in March to April 2024, with a total of 53 participants. The data collected included participants' sociodemographic profiles, oral conditions, and oral health-related quality of life (OHRQoL) measured with the Oral Health Impact Profile 14 (OHIP-14) questionnaire, which was adapted to Indonesian language.

**Statistical Analysis:**

Kendall and Spearman tests with a significance level of 5% were used to analyze the correlations between variables for bivariate analysis.

**Results:**

Study participants consisted of 32 males (60.4%) and 21 females (39.6%), with the majority (22 participants, 41.5%) aged 46 to 55 years old. Nasopharyngeal cancer was reported to be the most prevalent HNC diagnosis in this study at 69.8%. All study participants (100%) experienced xerostomia and had clinical oral dryness as their short-term radiotherapy-related oral complications. The distribution of OHIP-14 scores showed that most of the participants (73.6%) had moderate OHRQoL. Xerostomia and dysphagia were shown to have statistically significant influence on OHIP-14 score (Spearman test, *p* < 0.05).

**Conclusion:**

The majority of HNC patients treated with radiotherapy in Dharmais Cancer Hospital have moderate QoL. Dysphagia and xerostomia were reported as the primary drivers of reduced QoL in our study, indicating potential key targets for preventive and management strategies in HNC radiotherapy patients.

## Introduction

The Global Cancer Observatory (GLOBOCAN) 2020 data show that the number of new head and neck cancer (HNC) cases in Indonesia reached the number of 33,798 with a death toll of around 20,861. 1 High-dose radiation therapy for HNC can cause several side effects that may appear during or after radiotherapy. They are divided into two: acute/short-term effects that occur during radiotherapy or shortly (about 2 to 3 weeks) after radiotherapy is completed, and chronic/long-term effects that can manifest at any time from weeks to several years after radiotherapy is completed. 2 Short-term oral complications of radiotherapy include oral mucositis, decreased saliva secretion, infections, pain, and sensory disturbances. 2 3 4

The oral health-related quality of life (OHRQoL) is greatly affected by oral complications of radiotherapy. 5 6 Oral complications can impact the ability of speaking, mastication, and swallowing, leading to weight loss and a decrease in the patient's quality of life (QoL), thereby delaying the therapy process. 7 8 Studies on oral complications from head and neck radiotherapy have been reported, showing that there were differences in QoL score between groups of patients who regularly underwent dental checkups before, during, and after radiotherapy and those who did not, indicating that oral checkup and treatment were very important to achieve good OHRQoL values. 9 Certain short-term oral complications of radiotherapy can persist for weeks, months, or even become permanent after treatment ends. These issues can severely impact QoL and daily functioning, sometimes leading to long-term conditions if not managed properly. 2 8 However, research on short-term oral complications and their impact on OHRQoL is rarely reported globally and is currently unavailable in Indonesia. Therefore, the aim of this study was to describe and analyze the effect of short-term oral complications of head and neck radiotherapy on OHRQoL, to provide insights toward comprehensive care and improvement in the QoL of HNC patients after therapy.

## Materials and Methods

This was a descriptive-analytical study with a cross-sectional design with nonprobability sampling conducted for approximately 2 months at the Dharmais Cancer Hospital at Jakarta, Indonesia, which is a national referral hospital for cancer patients in the country. The research protocol was reviewed and granted by the Ethical Commission for Dental Research Faculty of Dentistry Universitas Indonesia (No. 8/Ethical Approval/FKGUI/II/2024) and the Committee of the Medical Research Ethics of Dharmais Cancer Hospital (No. DP.04.03/11.5/049/2024). Sample size was calculated using the G*Power program, resulting in a minimum number of 47 participants. 10

### Study Participant Inclusion Criteria

The inclusion criteria for this study were HNC patients (ICD 10: C00-C11) who were undergoing or had completed head and neck radiotherapy within <3 months of data collection, received radiotherapy, were ≥18 years old with good cognitive function and passed the Mini-Mental State Exam with scores of 25 to 30 for elderly respondents and were willing to be participants and signed the informed consent.

### Study Participants Exclusion Criteria

The exclusion criteria in this study were HNC patients with comorbidities (diabetes mellitus, Sjogren's syndrome, Parkinson's disease, autoimmune disorders, tuberculosis, sarcoidosis, amyloidosis, graft versus host disease, and HIV/AIDS), incomplete medical records, and prescribed with xerogenic drugs (such as sertraline, amitriptyline, clozapine, haloperidol, captopril, clonidine, biperiden, furosemide, morphine, atropine, codeine, methadone, tramadol, diazepam, diphenhydramine, omeprazole, lansoprazole, ranitidine, nifedipine, bisoprolol, cetirizine, gabapentin, levetiracetam, and carbamazepine).

### Data Collection

The data were collected from March to April 2024. The total number of recruited participants was 57, but four of them were eventually excluded due to a history of comorbidities. Thus, the final participants' number was 53. The data collected in this study included data from medical record (age, gender, therapy modality, radiotherapy dose, radiotherapy method, cancer stage, comorbidities, medication history, and oral infection elimination status), short-term oral complication associated with radiotherapy (oral mucositis, dysphagia, xerostomia, clinical oral dryness, hyposalivation, taste dysfunction, and candidiasis) evaluated by standardized examiners, and OHRQoL. The data collection form including the questionnaires written in Indonesian is available as Supplementary Data (available in online version only).

Oral mucositis was graded according to the World Health Organization Oral Mucositis Scale based on the mucositis severity. 11 The presence of dysphagia and taste dysfunction was recorded from the participants' direct report. Xerostomia refers to the subjective experience of dry mouth measured by interview using the Summated Xerostomia Inventory that has been adapted into Indonesian (SXI-ID). 12 This instrument consisted of five questions with 5-point Likert scale responses (possible total score 0–25). The participant was said to have no xerostomia if the total score was less than 6, mild xerostomia if the total score was between 6 to 15, moderate xerostomia if the score was between 16 to 20, and severe xerostomia if the score was more than 20.

The Clinical Oral Dryness Scale was used to evaluate oral dryness using 10 major signs of dry mouth. Each sign equally weighs 1 point; thus, the total findings can be interpreted mild (score 1–3), moderate (score 4–6), and severe (score 7–10) oral dryness. 13 Hyposalivation was objectively measured by calculating the unstimulated salivary flow rate (USFR). The participants were asked to let their saliva flowed into a graduated flask without any stimulation for 5 minutes. Hyposalivation was defined if the USFR was less than 0.1 mL/min, normal salivary flow rate was ≥0.3 mL/min, and 0.1 to <0.3 mL/min were defined as below normal USFR. 14

Participants were interviewed using the Indonesian version of the Oral Health Impact Profile 14 (OHIP-14) questionnaire to measure the OHRQoL. This questionnaire had also been translated and adapted for the Indonesian population. 15 The OHIP-14 questionnaire consists of 14 questions that represent seven domains, namely functional limitation, physical pain, psychological discomfort, physical, psychological, and social disability, and handicap.

Face validity was performed at the beginning of data collection on the first 10% of the minimum number of participants (10 persons). None of the participants reported any difficulty in understanding the questionnaire.

### Statistical Analysis

The normal distribution data were checked by using the Kolmogorov–Smirnov test. Univariate analysis in percentage and frequency distribution was presented for each data variable. The correlation test for the short-term oral complications and the OHIP-14 score was performed using the Spearman test to determine the strength and direction of the relationship between the variables. The statistical calculations were conducted using IBM SPSS 24 software (IBM Corp, Armonk, New York, United States).

## Results

All numerical data in this study had undergone a normality test with the result where the data were normally distributed.

The participants' profiles in this study are shown in Table 1 . Out of a total of 53 contributing participants, 32 were males (60.4%) and 21 were females (39.6%). The age groups in this study were divided into six groups, in which most participants were in the 46 to 55 age group, totaling 22 participants (41.5%). The most common cancer type among the participants was nasopharyngeal cancer at 69.8% (37 participants). Moreover, most participants (40 participants, 75.5%) were diagnosed with cancer stage IV, treated with chemotherapy and radiotherapy (37 participants, 69.8%), specifically using volumetric modulated arc therapy (VMAT) radiotherapy technique in 41 participants (77.4%).

**Table 1:** Study participants' profiles

Variable	*N* ( *N* = 53)	%
Sex		
Male	32	60.4
Female	21	39.6
Age group		
18–25 y	2	3.8
26–35 y	5	9.4
36–45 y	8	15.1
46–55 y	22	41.5
56–65 y	10	18.9
≥66 y	6	11.3
Cancer type		
Nasopharyngeal	37	69.8
Laryngeal	4	7.5
Tonsil	2	3.8
Tongue	2	3.8
Sinonasal	3	5.7
Palate	3	5.7
Oropharyngeal	2	3.8
Cancer stage		
Stage I	0	0
Stage II	4	7.5
Stage III	9	17
Stage IV	40	75.5
Treatment modalities		
Radiotherapy	2	3.8
Surgery and radiotherapy	6	11.3
Chemotherapy and radiotherapy	37	69.8
Surgery, chemotherapy, and radiotherapy	8	15.1
Radiotherapy technique		
Conventional 3D	3	5.7
IMRT	9	17
VMAT	41	77.4
Oral infection elimination status		
Not performed	6	11.3
Incomplete	5	9.4
Completed	42	79.2

Abbreviations: IMRT, intensity-modulated radiation therapy; VMAT, volumetric-modulated arc therapy.

Most of the participants had completed the oral infection elimination (79.2%). However, there were five participants (9.4%) who had not completed the oral infection elimination and six participants (11.3%) who did not receive oral checkup by a dentist before radiotherapy.

The distribution of short-term oral complications of head and neck radiotherapy in this study is shown in Table 2 . The top prevalences of the oral complications were taste dysfunction in 46 participants (86.8%), followed by hyposalivation in 40 participants (75.5%), dysphagia in 38 participants (71.7%), severe xerostomia in 37 participants (69.8%), and moderate clinical oral dryness in 36 participants (67.9%).

**Table 2:** Distribution of short-term oral complications ( *N* = 53)

Oral complications	*N*	%
Oral mucositis		
None Grade 1 Grade 2 Grade 3 Grade 4	32	60.4
	13	24.5
	6	11.3
	2	3.8
	0	0
Dysphagia		
None Present	15	28.3
	38	71.7
Taste dysfunction		
None Present	7	13.2
	46	86.8
Oral candidiasis		
None Present	33	62.3
	20	37.7
Clinical oral dryness		
None	0	0
Mild Moderate Severe	3	5.7
	36	67.9
	14	26.4
Salivary flow		
None (normal) Below normal Hyposalivation	0	0
	13	24.5
	40	75.5
Xerostomia		
None Mild Moderate Severe	0	0
	4	7.5
	12	22.6
	37	69.8

The OHIP-14 questionnaire consists of seven domains, each domain consisted of two questions, 14 questions in total. Table 3 shows the frequency distribution of OHIP-14 scores among 53 research participants. The average OHIP-14 score was 23.87 ± 8.24. The highest average score was found in the physical disability domain at 5.58 ± 2.08, followed by the physical pain, psychological discomfort, and functional limitation domains with average values of 4.47 ± 1.66, 4.08 ± 2.15, and 4.06 ± 1.78, respectively.

**Table 3:** Distribution of OHIP-14 scores based on question items ( *N* = 53)

OHIP-14	*N* (%)					Mean ± SD
	0	1	2	3	4	
Functional limitation						4.06 ± 1.78
1. Have you had trouble pronouncing any words because of problems with your teeth or mouth?	31 (58.5)	3 (5.7)	12 (22.6)	3 (5.7)	4 (7.5)	0.98 ± 1.32
2. Have you felt that your sense of taste has worsened because of problems with your teeth or mouth?	5 (9.4)	2 (3.8)	8 (15.1)	7 (13.2)	31 (58.5)	3.08 ± 1.32
Physical pain						4.47 ± 1.66
3. Have you had painful aching in your mouth?	20 (37.7)	9 (17)	13 (24.5)	4 (7.5)	7 (13.2)	1.42 ± 1.40
4. Have you found it uncomfortable to eat any foods because of problems with your teeth or mouth?	2 (3.8)	2 (3.8)	13 (24.5)	10 (18.9)	26 (49.1)	3.06 ± 1.11
Psychological discomfort						4.08 ± 2.15
5. Have you been self-conscious because of your teeth or mouth?	2 (3.8)	8 (15.1)	12 (22.6)	13 (24.5)	18 (34)	2.70 ± 1.20
6. Have you felt tense because of problems with your teeth or mouth?	20 (37.7)	9 (17)	12 (22.6)	8 (15.1)	4 (7.5)	1.38 ± 1.33
Physical disability						5.58 ± 2.08
7. Has your diet been unsatisfactory because of problems with your teeth or mouth?	1 (1.9)	4 (7.5)	7 (13.2)	13 (24.5)	28 (52.8)	3.19 ± 1.05
8. Have you had to interrupt meals because of problems with your teeth or mouth?	8 (15.1)	7 (13.2)	12 (22.6)	8 (15.1)	18 (34)	2.40 ± 1.45
Psychological disability						1.98 ± 1.95
9. Have you found it difficult to relax because of problems with your teeth or mouth?	18 (34)	10 (18.9)	11 (20.8)	7 (13.2)	7 (13.2)	1.53 ± 1.42
10. Have you been a bit embarrassed because of problems with your teeth or mouth?	41 (77.4)	6 (11.3)	2 (3.8)	2 (3.8)	2 (3.8)	0.45 ± 1.01
Social disability						0.64 ± 1.41
11. Have you been a bit irritable with other people because of problems with your teeth or mouth?	45 (84.9)	1 (1.9)	4 (7.5)	1 (1.9)	2 (3.8)	0.38 ± 0.98
12. Have you had difficulty doing your usual jobs because of problems with your teeth or mouth?	46 (86.8)	2 (3.8)	3 (5.7)	2 (3.8)	0 (0)	0.26 ± 0.73
Handicap						3.06 ± 1.2
13. Have you felt that life in general was less satisfying because of problems with your teeth or mouth?	1 (1.9)	6 (11.3)	8 (15.1)	18 (34)	20 (37.7)	2.94 ± 1.08
14. Have you been totally unable to function because of problems with your teeth or mouth?	50 (94.3)	1 (1.9)	1 (1.9)	1 (1.9)	0 (0)	0.11 ± 0.50
Total				23.87 ± 8.24		

Abbreviations: OHIP-14, Oral Health Impact Profile 14; SD, standard deviation.

Table 4 shows the category of OHIP-14 scores. The scores were divided into three categories: good (0–18), moderate (19–37), and poor (38–56). Most of the participants had moderate QoL (39 participants, 73.6%), followed by good QoL (11 participants, 20.7%), and only 3 participants (5.7%) had poor QoL. Analysis of which oral complications affecting patients' OHIP-14 scores was shown in Table 5 using the Spearman test. Dysphagia and xerostomia showed significant and strong correlation toward OHIP-14 score ( *p* < 0.05; ρ 0.432 and 0.431, respectively) of the participants.

**Table 4:** Distribution of OHIP-14 scores ( *N* = 53)

OHIP-14 score category	*N* (53)	%
Good (0–18)	11	20.7
Moderate (19–37)	39	73.6
Poor (38–56)	3	5.7

Abbreviations: OHIP-14, Oral Health Impact Profile 14; SD, standard deviation.

**Table 5:** Analysis of effects of short-term oral complications on OHIP-14 score

Oral complication	OHIP-14 score	
	*p*	*ρ* (rho)
Oral mucositis	0.348	0.131
Dysphagia	0.001 a	0.432 b
Taste dysfunction	0.245	0.162
Oral candidiasis	0.079	0.243
Clinical oral dryness	0.734	−0.48
Hyposalivation	0.546	−0.085
Xerostomia	0.001 a	0.431 b

Abbreviations: OHIP-14, Oral Health Impact Profile 14; SD, standard deviation.

a Significant correlation, *p* < 0.05.

b Coefficient correlation strength.

## Discussion

The aim of the study was to examine the effect of short-term oral complications of head and neck radiotherapy on OHRQoL to gain insight on how comprehensive care and improvement in the QoL of HNC patients after therapy can be provided. Oral complications caused by head and neck radiotherapy can greatly affect the OHRQoL. They can impact the ability of speaking, masticating, and swallowing, which could lead to weight loss and a decrease in the patient's QoL, thereby delaying the therapy process. 5 16

The participants of this study were mostly males. This was consistent with various other studies that reported that HNC was significantly more common in males than in females. 17 18 The high risk of HNC in males can be attributed to the high rates of tobacco and alcohol consumption among them. 19 Tobacco and alcohol contain several carcinogenic substances that can cause damage to DNA cells and subsequently induce cancer. 17 20 21 Other risk factors related to HNC are betel nut chewing, especially in South, Southeast, and Polynesia regions and viral infection such as human papillomavirus, and Epstein–Barr virus (EBV) that were increasingly reported in younger populations due to changes in lifestyle. 17

This study shows that nasopharyngeal cancer was the most common type of HNC at Dharmais Cancer Hospital. These results were consistent with the data from GLOBOCAN 2022. 22 Most participants in this study were in stage IV, accounting for 75.5%, which aligns with the findings of To'Bungan et al. at Dharmais Cancer Hospital in 2015 saying that this situation could be caused by a lack of awareness and knowledge among the community to perform early detection and seek immediate treatment. 19 Similar findings were also reported in Uganda and Ethiopia that most individuals with HNC received delayed diagnosis due to poor educational and income levels, as well as restricted access to medical facilities. 23 24

Most participants received a combination therapy of chemotherapy and radiotherapy (chemoradiation), similar to the findings of Fekadu et al. 25 Radiotherapy plays an important role in the treatment of HNC, both as the primary and sole treatment modality or as a combination therapy with other treatment options. 26 27 28 The majority of radiotherapy methods used in this study were VMAT methods followed by intensity-modulated radiotherapy (IMRT). The VMAT method is reported to improve the efficiency of radiation delivery, enhancing therapy outcomes, reducing intrafraction motion, and minimizing side effects. According to a study by Algouneh et al., patients with HNC have a higher long-term survival rate using the VMAT technique than with IMRT. 29 However, more research is required because no study has yet compared the toxicity of the VMAT and IMRT procedures.

Dental and oral examinations before radiotherapy aim to eliminate infections in the oral cavity to prevent local, regional, or systemic complications during and after cancer treatment or other medical treatments. 16 30 31 In this study, the majority of participants had completed the oral infection elimination procedures before radiation therapy began because this procedure has been included in the Clinical Practice Guidelines (CPGs) at Dharmais Cancer Hospital before the start of radiation therapy. This guideline states that every HNC patient undergoing therapy—whether surgical, chemotherapy, or radiotherapy—must undergo examination and evaluation by a dentist before, during, and after therapy. Before cancer therapy, patients would receive oral infection elimination procedures and fluoride application. During cancer therapy, patients with oral complications will be referred to a dentist. Whereas after cancer therapy, patients will have routine checkups with the dentist, specifically in the first week, first month, and 3 months after the cancer therapy was completed. The CPGs at Dharmais Cancer Hospital are in accordance with the guidelines from the National Cancer Institute. 32 The same guideline has also been implemented in countries like Japan and South Korea. Previous studies by Sohn et al. and Nibu et al. reported that professional oral care and dental examination before and after HNC therapy was beneficial for preserving oral health in patients with HNC. 33 34

High-dose radiation therapy to areas such as the oral mucosa, skin, maxilla, mandible, and salivary glands can cause several unwanted oral complications that may appear during or after the therapy is completed. 35 36 These oral complications are divided into two types: short-term (acute) and long-term (chronic). 2 In this study, we started by collecting short-term oral complications such as xerostomia, clinical oral dryness, taste dysfunction, oral mucositis, dysphagia, oral candidiasis, and hyposalivation. Our subsequent study will report the long-term oral complications in this participant cohort.

Radiation can cause damage to the salivary gland cells, resulting in fibrosis, reduced salivary flow rate, and changes in saliva composition. 37 This study showed that all participants experienced xerostomia and clinical oral dryness. Moreover, objective hyposalivation was reported in 75.5% of participants. These results were strengthened by what Palmieri et al. had previously reported that by the fourth week of radiotherapy, all participants experienced xerostomia complaints of varying degrees, and the complaints worsened over the course of the radiotherapy, peaking from the second to the third week. 38 Additionally, the study by Abou-Bakr et al., reported that higher prevalence of late xerostomia and hyposalivation in HNC patients was significantly correlated with tumor site, greater radiation doses, and concurrent chemoradiotherapy leading to long-term consequences such as decreased unstimulated salivary flow. 39

Taste dysfunction occurred in 86.8% of participants in this study. This result was similar to the study conducted by Mathlin et al., which reported that 97% of participants complained of taste disturbances in the fourth week after radiation therapy. 40 Radiotherapy can cause dysgeusia due to changes in the structure of the palatal pores and thinning of the papilla epithelium. A significant decrease in taste function is associated with a reduction in saliva flow rate, which can decrease the transport and solubility of gustatory stimuli, leading to an inability to taste. These disorders significantly impact appetite and dietary patterns, leading to weight loss, nutritional deficiencies, and a decrease in the patient's QoL. 40 41 42

One of the oral complications that upset patients was dysphagia that occurs because of radiation damage to the tissues, nerves, and from fibrosis or scar tissue in the physiological structures related to swallowing function. Additionally, dysphagia can also occur as a result of other oral complications such as mucositis, dry mouth, or oral candidiasis. Dysphagia directly involved in patients' food intake changes that might lead to weight loss and malnutrition. 43 44 In this study, 71.7% of participants had dysphagia complications. This result was in line with the research conducted by Alexidis et al., which found that the average percentage of head and neck patients suffering from dysphagia was 40 to 70%. 45 This disorder might require patients to use a nasogastric tube (NGT) to help them to meet their nutritional intake.

Oral mucositis has been reported to be the most common complication of radiotherapy, beginning with acute inflammation, extensive ulcers, and finally pain in the oral mucosa and oropharynx. This condition can cause dysphagia, nutritional problems, fungal infections, and often disrupting the therapy process and leading to delays in radiotherapy. 46 47 48 In radiotherapy patients, higher salivary oxidative stress has been shown to be positively correlated with the severity of oral mucositis. 49

As many as 39.6% of participants in this study suffered from mucositis, ranging from grade 1 to grade 3. This result differed from several literature, which stated that radiotherapy-induced oral mucositis can occur in approximately >80% of patients. 48 50 This difference may occur because the majority of participants in this study had completed radiotherapy more than 1 month prior to the data collection, which, according to the study by Sroussi et al. and Maria et al., showed that oral mucositis usually healed within 2 to 4 weeks after completion of the radiotherapy or chemotherapy. 46 50 Another possibility is the radiotherapy methods applied to the majority of the participants in this study, both IMRT and VMAT, provided important benefits by enabling accurate radiation delivery, lowering toxicity, and possibly reducing treatment duration. Specifically, VMAT provided better efficiency and possibly better dose conformance than conventional IMRT. 51 Recent clinical trials by Hassanein et al. reported that amino acid-based therapy, specifically L-arginine and L-glutamine, effectively reduced the severity of the oral mucositis, alleviated discomfort, and helped maintain body weight, thus enhancing OHRQoL in patients with HNC. 52

Oral candidiasis was found in 37.7% participants of this study, established through clinical examination. The prevalence of oral candidiasis in several studies ranges from 17 to 52%. 49 Radiotherapy can cause damage to the mucosal barrier and facilitate microbial colonization and infection, leading to the expansion of tissue damage. 53 The data on oral candidiasis in this study were relatively low because the CPGs at Dharmais Cancer Hospital included prescription of topical antifungals and chlorhexidine mouthwash to patients undergoing radiotherapy to prevent oral candidiasis.

Research by Tesic et al. shows that OHIP-14 is valid and reliable for use in HNC patients. 54 The mean of OHIP-14 score in this study was 23.87, and most participants in this study have moderate QoL based on their OHIP-14 scores. This result was similar with the study conducted by Suarantari and Winata, which reported that their OHIP-14 mean score was 21.47, and most patients had moderate QoL. 55 Patients who have oral complications during and after radiotherapy can experience impaired function of the oral cavity, stress, and generally lost their appetite, causing nutritional deficiencies that can affect their QoL. 56

Lastly, our current study had several limitations that might restrict the analysis of the results. The inclusion and exclusion criteria in this study were too restrictive and the participants were only recruited from one hospital within a short data collection period of approximately 2 months, resulting in small final number of participants that might not be representative of all patients or broader health care landscape since the CPGs can differ among hospitals. Nevertheless, these limitations created a homogenous sample, which enhanced the internal validity of this study.

## Conclusion

This study was the first in Indonesia to collect data on all short-term oral complications of HNC radiotherapy and analyze their effects on the OHRQoL. The study results showed that the most commonly found short-term oral complications were dysphagia, taste dysfunction, xerostomia, clinical oral dryness, and abnormal salivary flow rate. The result of this study also showed that OHIP-14 score was significantly affected by dysphagia and xerostomia, suggesting these variables as the primary drivers of reduced QoL. Providing adequate management and prevention of these conditions might potentially improve HNC patients' QoL, especially after radiotherapy.
