# Supplementary material for: Head and Neck Radiotherapy Short-Term Oral Complications: Effect on Oral Health-Related Quality of Life at Dharmais Cancer Hospital
Source: Eur J Dent. 2026 Feb 12;20(4):1273–80. doi: 10.1055/s-0046-1816079 (PMC13623472; doi:10.1055/s-0046-1816079)
Supplement: Supplementary file 1 [file 10-1055-s-0046-1816079-s2594487.pdf]

KUESIONER DAN LEMBAR PEMERIKSAAN PENELITIAN

I. Profil Subjek Penelitian

Nama* :
Jenis Kelamin :
Tanggal lahir/usia :
Suku/Ras* :
Alamat :
No. Tlp* :
Pendidikan* :
Pekerjaan* :
Riwayat Medis : **(beri tanda √)**

- ○ Diabetes melitus
- ○ Sindrom sjogren
- ○ Parkinson
- ○ Kelainan autoimun
- ○ Tuberkulosis
- ○ Sarkoidosis
- ○ Amilodosis
- ○ Graft versus host disease
- ○ HIV/AIDS
- ○ Lainnya: ....

Riwat Penggunaan Obat* :
Jenis Kanker :
Staging Kanker : **(beri tanda √)**

- ○ Stadium I
- ○ Stadium II
- ○ Stadium III
- ○ Stadium IV

Modalitas Terapi : **(beri tanda √)**

- ○ Radioterapi
- ○ Radioterapi & Bedah
- ○ Radioterapi & Kemoterapi
- ○ Radioterapi, Bedah & Kemoterapi

Metode Radioterapi : **(beri tanda √)**

- ○ 2D Konvensional
- ○ 3D Konvensional
- ○ IMRT

Dosis Radioterapi :
Status Eliminasi FI : **(beri tanda √)**

- ○ Tidak sama sekali
- ○ Belum Selesai
- ○ Selesai

Indeks DMF-T Praradioterapi :

II. Kuesioner OHIP-14 Versi Bahasa Indonesia)*

Nama:						
Pertanyaan	Seberapa sering anda mengalami masalah ini dalam 1 tahun terakhir?					
	Jawaban					
	Sangat sering	Sering	Kadang-kadang	Sangat Jarang	Tidak pernah	Tidak tahu
1. Pernahkah anda kesulitan dalam mengucapkan kata-kata, karena ada masalah dengan gigi dan mulut atau gigi tiruan anda?						
2. Pernahkan merasakan indra pengecapan anda menjadi buruk karena masalah gigi, mulut dan gigi tiruan anda?						
3. Pernahkah anda mengalami sakit yang amat sangat dalam mulut anda?						
4. Pernahkan anda merasa tidak nyaman saat makan karena masalah pada gigi, mulut atau gigi tiruan?						

(*Continued*)

 © 2026. The Author(s).

(*Continued*)

**Nama:**						
**Pertanyaan**	**Seberapa sering anda mengalami masalah ini dalam 1 tahun terakhir?**					
	**Jawaban**					
	**Sangat sering**	**Sering**	**Kadang-kadang**	**Sangat Jarang**	**Tidak pernah**	**Tidak tahu**
5. Pernahkah anda merasa risih / tidak nyaman karena gigi, mulut atau gigi tiruan anda?						
6. Pernahkah anda merasa tertekan karena masalah pada gigi, mulut atau gigi tiruan?						
7. Pernahkan merasa makanan anda tidak memuaskan karena masalah gigi, mulut atau gigi tiruan?						
8. Pernahkah anda harus berhenti sebentar saat makan karena masalah gigi, mulut atau gigi truan?						
9. Pernahkah anda merasa sulit rileks karena masalah dengan gigi, mulut atau gigi tiruan?						
10. Pernahkah anda merasa agak malu karena masalah gigi, mulut atau gigi tiruan?						
11. Pernahkah anda mudah tersinggung karena masalah gigi, mulut atau gigi tiruan?						
12. Pernahkah anda kesulitan melakukan kegiatan sehari-hari karena masalah gigi, mulut atau gigi tiruan?						
13. Pernakah merasa kurang puas dengan kehidupan anda karena masalah gigi, mulut atau gigi tiruan?						
14. Pernahkah anda sama sekali tidak bisa berkegiatan karena masalah gigi, mulut atau gigi tiruan?						

III. Pemeriksaan Sialometri dan SXI-ID*

A. *Summated Xerostomia Inventory Indonesian Version* (SXI-ID)

Skoring: 1 = tidak pernah, 2 = hampir tidak pernah, 3 = kadang-kadang, 4 = sering, 5 = selalu

1. Apakah mulut anda terasa kering?	
2. Apakah anda kesulitan makan makanan yang kering?	
3. Apakah mulut anda terasa kering saat sedang makan?	
4. Apakah anda kesulitan menelan makanan tertentu?	
5. Apakah bibir anda terasa kering?	
**Total skor**	

*Hampir tidak pernah : 1-2 kali dalam sebulan
*Kadang-kadang : 1 kali dalam seminggu
*Sering : 2-3 kali dalam seminggu
**Makanan kering (pertanyaan 2) : kerupuk, gorengan, kue kering
**Makanan tertentu (pertanyaan 4) : roti, dodol, kue kering

 © 2026. The Author(s).

B. *Clinical Oral Dryness Score* (CODS)
Skoring 0 = Tidak ada, 1 = Ada

1. Kaca mulut menempel pada mukosa bukal	
2. Kaca mulut menempel pada lidah	
3. Lidah berlobus-lobus/berfisur	
4. Kehilangan papilla lidah	
5. Saliva berbuih	
6. Tidak ada pooling saliva di dasar mulut	
7. Mukosa oral lainnya terlihat mengkilat, terutama palatum	
8. Debris menempel pada palatum (tidak termasuk debris di bawah gigi tiruan)	
9. Perubahan arsitektur gingiva atau gingiva halus (*loss of stippling*)	
10. Karies servikal aktif atau baru saja direstorasi dalam 6 bulan terakhir, > 2 gigi	
**Total skor**	

C. *Unstimulated Salivary Flow Rate* (USFR)
Hasil ukur : ...... ml/menit

Normal : $\geq$0,3 ml/menit
Di bawah normal : 0,1 - <0,3 ml/menit
Hiposalivasi : <0,1 ml/menit

IV. Indeks DMF-T
DECAY, MISSING, FILLED TEETH

18	28
17	27
16	26
15	25
14	24
13	23
12	22
11	21

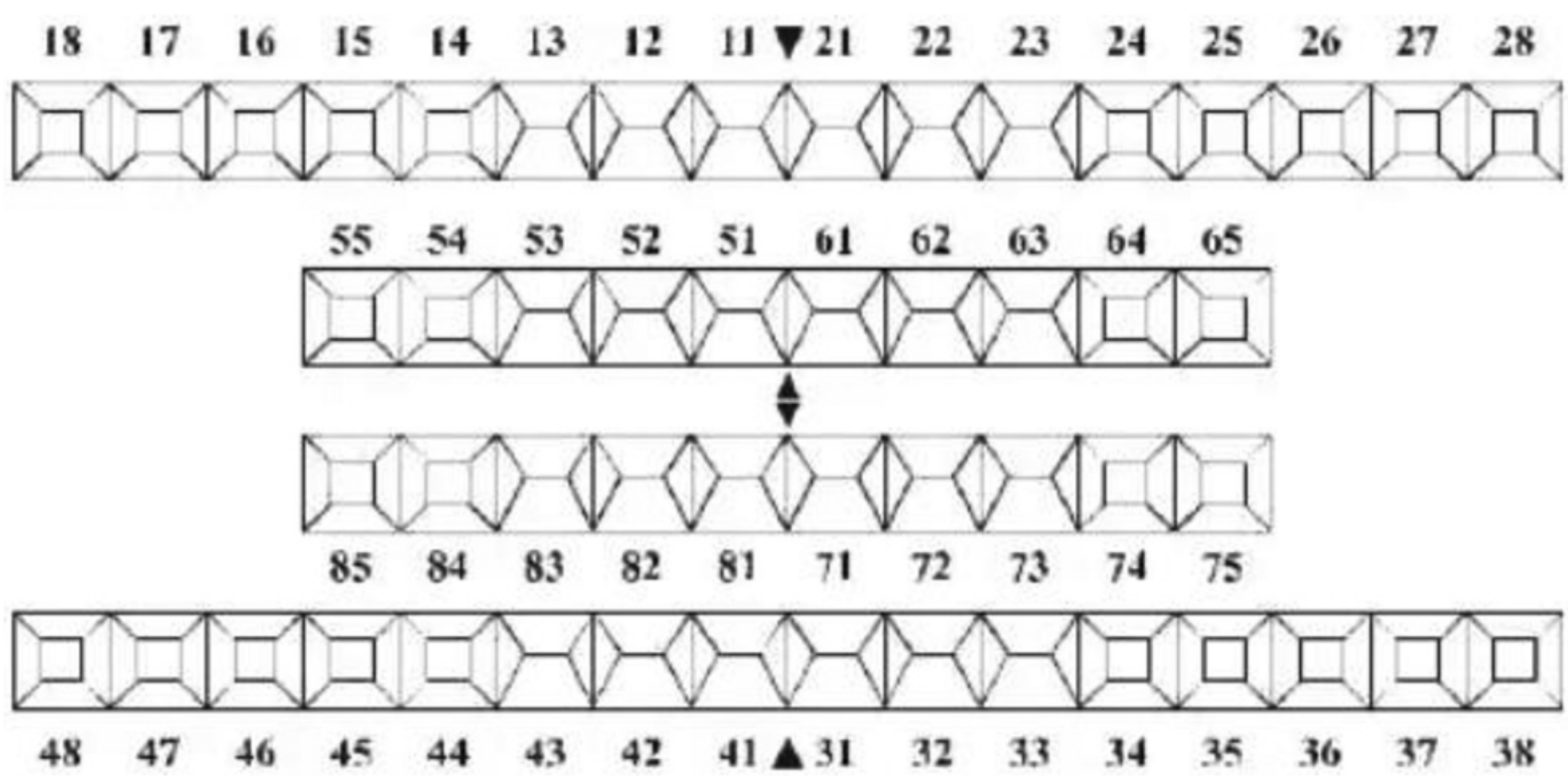

 © 2026. The Author(s).

41	31
42	32
43	33
44	34
45	35
46	36
47	37
48	38

Decay =
Missing =
Filled =
DMF-T =

V. Komplikasi Oral Radioterapi (beri tanda √)

Mukositis Oral	○ Tidak ada<br>○ Derajat 1: Nyeri dan eritema.<br>○ Derajat 2: Eritema dan ulserasi, pasien dapat menelan makanan solid.<br>○ Derajat 3: Ulserasi dengan eritema yang luas, pasien tidak dapat menelan makanan.<br>○ Derajat 4: Ulserasi yang luas, fungsi oral tidak memungkinkan.
Disfagia	○ Tidak Ada<br>○ Ada
Disfungsi Pengecapan	○ Tidak ada<br>○ Ada
Kandidiasis	○ Tidak ada<br>○ Ada
Mulut Kering (*Oral Dryness*)<br>*Menggunakan instrument C*linical Oral Dryness Score* (CODS)	○ Tidak ada<br>○ Ringan (Skor 1-3)<br>○ Sedang (Skor 4-6)<br>○ Berat (Skor 7-10)
Xerostomia<br>*Menggunakan kuesioner *Summated Xerostomia Inventory Indonesian Version* (SXI-ID)	○ Tidak xerostomia (Skor 5)<br>○ Xerostomia ringan (Skor >5-15)<br>○ Xerostomia sedang (Skor >15–20)<br>○ Xerostomia berat (Skor >20-25)
Hiposalivasi<br>*Sialometri menggunakan *Unstimlate Saliva Flow Rate* (USFR) dengan *spitting method*	○ Normal (≥ 0.3 ml/menit)<br>○ Dibawah normal (0,1 - <0.3 ml/menit)<br>○ Hiposalivasi (< 0.1 ml/menit
Oral Health Related Quality of Life (OHRQoL)<br>*Menggunakan kuesioner OHIP-14	Skor: ....

 © 2026. The Author(s).
